# Factors Associated With Persisting Symptoms After Concussion in Adults With Mild TBI: A Systematic Review and Meta-Analysis

**DOI:** 10.1001/jamanetworkopen.2025.16619

**Published:** 2025-06-18

**Authors:** Samantha J. McIntosh, Melanie H. Vergeer, Jean-Michel Galarneau, Paul H. Eliason, Chantel T. Debert

**Affiliations:** 1 Department of Clinical Neurosciences, Cumming School of Medicine, University of Calgary, Calgary, Alberta, Canada; 2 Hotchkiss Brain Institute, University of Calgary, Calgary, Alberta, Canada; 3 Sport Injury Prevention Research Centre, Faculty of Kinesiology, University of Calgary, Calgary, Alberta, Canada

## Abstract

**Importance:**

Up to 30% of individuals who sustain a mild traumatic brain injury (mTBI) develop persisting symptoms after concussion (PSAC). Identifying acute risk factors for PSAC can enhance clinical care in adults with concussion.

**Objective:**

To evaluate summary odds ratio (OR) estimates for acute factors associated with PSAC in adults with mTBI.

**Data Sources:**

For this systematic review and meta-analysis, a systematic search of Ovid MEDLINE, Embase, PsycINFO, CINAHL, SPORTDiscus, and the Cochrane Central Register of Clinical Trials (studies published from 1970 to February 15, 2024) and backward reference searching was performed on February 15, 2024. Search terms were *mTBI*, *concussion*, *prognostic variables*, *predictors*, and *PSAC.*

**Study Selection:**

Peer-reviewed studies in English that reported clinical factors collected within 1 month (≤28 days) of injury and associated with poor outcome, specifically PSAC, more than 1 month after concussion were included. The Preferred Reporting Items for Systematic Reviews and Meta-Analyses guideline was followed.

**Data Extraction and Synthesis:**

Independent extraction and quality assessment were performed by 2 author reviewers. Study characteristics and ORs were extracted using the Modified Checklist for Critical Appraisal and Data Extraction for Systematic Reviews of Prognostic Factor Studies. Risk of bias was assessed using the Quality in Prognostic Studies tool. Three-level meta-analytic models examined factors associated with PSAC 1, 3, and 6 months after mTBI.

**Main Outcomes and Measures:**

Adjusted OR (AOR) estimates for the association of acute clinical measures with PSAC.

**Results:**

Thirty-four studies were reviewed; data were extracted from 15 studies (44.1%) included in the meta-analysis (592 406 adults with concussion; mean age, 29.3 years [range, 16-89 years]; 57.8% male). Acute concentration difficulty was associated with greatest odds of PSAC across all time points (AOR, 3.43 [95% CI, 1.85-6.36]). Medical history of anxiety and/or depression or of sleep disorders (AOR, 2.47 [95% CI, 1.62-3.78]) and clinical signs (loss of consciousness and amnesia; AOR, 1.90 [95% CI, 1.28-2.84]) were associated with increased odds of PSAC across all time points. At 1 month (AOR, 3.12 [95% CI, 1.43-6.82]) and 6 months (AOR, 26.81 [95% CI, 3.42-210.06]), difficulty concentrating was associated with greatest odds of PSAC, while medical history of anxiety and/or depression or of sleep disorders was associated with greatest odds of PSAC at 3 months (AOR, 2.92 [95% CI, 1.39-6.14]).

**Conclusions and Relevance:**

In this systematic review and meta-analysis, acute cognitive symptoms, medical history of anxiety and/or depression or of sleep disorders, and the presence of clinical signs (loss of consciousness and amnesia) were the factors associated with greatest odds of PSAC in adults across all time points. The findings suggest that evaluating specific acute symptoms and signs may contribute to the prognosis of PSAC in adults.

## Key Points

**Question:**

What acute clinical measures are associated with persisting symptoms after concussion (PSAC) in adults with mild traumatic brain injury?

**Findings:**

In this systematic review and meta-analysis of 15 studies including 592 406 adults, factors associated with greatest overall odds of PSAC varied 1, 3, and 6 months after concussion. Factors associated with greatest odds across all time points were acute cognitive symptoms (difficulty concentrating), premorbid anxiety and/or depression or sleep disorders, and clinical signs (loss of consciousness and amnesia).

**Meaning:**

These findings suggest that evaluation of specific acute symptoms and signs may contribute to the prognosis of PSAC in adults.

## Teaser

This systematic review and meta-analysis explores whether specific acute clinical factors are associated with the development of persisting symptoms after a concussion or mild traumatic brain injury (TBI) in adults.

## Introduction

Approximately 70 million individuals sustain a traumatic brain injury (TBI) annually; of these TBIs, 80% are mild TBIs (mTBIs), commonly known as concussions.^1,2^ Concussion symptoms (headache, dizziness, nausea, and vomiting) typically resolve within 30 days.^3^ However, up to 30% of adults experience prolonged symptoms, termed *persisting symptoms after concussion* (PSAC),^4^ or *persisting postconcussion symptoms*; PSAC can negatively impact quality of life and prolong return to activities of daily living.^5,6,7,8^ Accurate prognosis for concussion recovery remains a high priority to improve concussion care.^9^

A recent cross-sectional survey of individuals with concussion, caregivers, and clinicians highlighted that identifying factors predicting prolonged recovery is the foremost unanswered research question in the field.^9^ The goal of identifying prognostic factors for PSAC is to develop tailored strategies to manage symptoms and support recovery. A standardized clinical tool to identify individuals at risk of PSAC could facilitate referral to specialized resources. However, there is currently no widely used prognostic tool for adults with concussion.

Synthesis of risk factors is necessary to develop an accurate prognostic tool. Thus, the primary aim of this systematic review and meta-analysis was to identify acute clinical factors associated with PSAC in adults with mTBI. Previous systematic reviews examining prognostic factors for PSAC lacked generalizability in an adult cohort with acute concussion due to recruitment location, population of interest, time after injury, and TBI severity.^10,11,12,13^ To our knowledge, this is the first meta-analysis of acute clinical factors associated with PSAC in adults with mTBI and will contribute to the literature by synthesizing current evidence on the most prominent factors associated with PSAC in this population.

## Methods

### Protocol and Registration

This systematic review and meta-analysis followed the Preferred Reporting Items for Systematic Reviews and Meta-Analyses (PRISMA) reporting guideline.^14^ The prespecified protocol was published on PROSPERO.

### Data Sources and Search Strategy

Ovid MEDLINE, Embase, PsycINFO, CINAHL, SPORTDiscus, and the Cochrane Central Register of Controlled Trials were searched on February 15, 2024, for studies published from 1970 to February 15, 2024. The search strategy was developed with the assistance of a health sciences librarian (eTable 1 in Supplement 1). Search terms were *mTBI*, *concussion*, *prognostic variables*, *predictors*, and *PSAC.* Additional backward reference searching was performed. Records were directly exported into Covidence systematic review software, version 2024 (Covidence Inc), where duplicates were removed.

### Eligibility Criteria and Study Selection

eTable 2 in Supplement 1 provides detailed eligibility criteria. Studies were included if clinical factors were collected within 1 month (≤28 days) after concussion and were associated with a poor outcome (eg, PSAC or persisting postconcussion symptoms, prolonged return to work) more than 1 month (>28 days) after concussion. We included original, full-text research published in English in peer-reviewed journals. Two reviewers (S.J.M., M.H.V.) independently screened titles, abstracts, and full texts in duplicate using Covidence. Discrepancies at any stage were discussed and resolved by consensus or by a third reviewer (C.T.D.).

### Data Extraction and Quality Assessment

Data extraction and quality assessment were performed independently by 2 reviewers (S.J.M., M.H.V.). Data were extracted according to the Modified Checklist for Critical Appraisal and Data Extraction for Systematic Reviews of Prognostic Factor Studies.^15^ Study authors were contacted to request missing or clarifying information. Risk of bias was assessed as high, moderate, or low as guided by the Quality in Prognostic Studies tool.^16,17^

### Statistical Analysis

We performed a meta-analysis of studies examining PSAC as the primary outcome. Factors associated with PSAC from single studies were combined in meta-analytic models accounting for situations of dependent effect sizes and were examined by time point of interest (1, 3, and 6 months after concussion).^18^ Estimates shown are adjusted odds ratios (AORs) with their corresponding 95% CIs produced by a restricted maximum-likelihood, 3-level (fixed effects, study, and factors) random-intercept model with the fixed effects fitted with a time × factor interaction^18^; this allowed for time-stratified estimated overall effect estimates for each factor while retaining the ability to display results from each study. Overall heterogeneity was quantified with the Cochran *I*^2^ statistic, and homogeneity was assessed with the Cochran *Q* statistic. The Higgins-Thompson *I*^2^ statistic was used to quantify level-specific heterogeneity of the random-effects model. Average marginal effect estimates for each factor across time points were estimated. Where possible, potential modification of associations was examined by visually comparing the overall effect estimates for common factors across time points but was not statistically tested given that specific studies were not included at each time point. Data were analyzed using Stata, version 18.0 (StataCorp LLC).

## Results

We retrieved 8564 records, of which 2329 were duplicates. In total, 5657 abstracts were screened and 575 full-text articles were assessed; 34 studies were included in the systematic review (eFigure 1 in Supplement 1).^19,20,21,22,23,24,25,26,27,28,29,30,31,32,33,34,35,36,37,38,39,40,41,42,43,44,45,46,47,48,49,50,51,52^ Nineteen studies (55.9%) examined single or multiple factors associated with PSAC^19,20,21,22,23,24,25,26,27,28,29,30,31,32,33,34,35,36,37^; 4 of those (21.1%) examined unique factors not reported in more than 1 study and were excluded from the meta-analysis.^19,20,21,22^ Thus, 15 studies (44.1%) were included in the meta-analysis examining factors associated with PSAC (Table).^23,24,25,26,27,28,29,30,31,32,33,34,35,36,37^ Studies excluded from the meta-analysis examined symptom severity,^38,39,40,41,42,43,44,45^ time to symptom resolution,^46,47^ return to work,^48^ posttraumatic stress disorder,^38,49^ satisfaction with or quality of life,^39,40^ clinician visits,^50^ activity levels,^51^ or physician-defined poor outcome^52^ (eTable 3 in Supplement 1).

**Table.** Characteristics of Studies Included in Meta-Analysis

Source	Country	Setting	Design	No. with mTBI	Age, y	Females, %	No. at follow-up	No. with PSAC	mTBI diagnostic criteria	PSAC diagnostic criteria
**1 Month**										
Bazarian et al,^25^ 1999	US	ED	Prospective case-control	71	Mean, 29.0	49.3	69	40	GCS 15, LOC <10 min, no abnormal CT findings	*DSM-IV*
Eskridge et al,^34^ 2013	US	Military (EMED)	Retrospective medical record review	1656	Mean (SD), 24.1 (4.9)	0	1656	254	*ICD-9* codes	*ICD-9* codes
Meehan et al,^33^ 2016	US	mTBI clinic	Prospective cohort	64	Mean (SD), 21.0 (2.0)	46.7	64	24	Consensus on Concussion in Sport	≥1 Symptom on PCSC
Mehrolhassani et al,^26^ 2020	Iran	ED	Prospective cohort	364	Median, 30.0 (range, 16-89)	60.4	364	120	Physician diagnosed, GCS 13-15	≥3 Symptoms (*ICD-10*)
Rowe et al,^35^ 2022	Canada	ED	Prospective cohort	248	Median, 35 (IQR, 23-49)	52.4	220	134	WHO	≥1 Symptom, ≥2 severity on RPQ
Varner et al,^37^ 2021	Canada	ED	Secondary analysis of RCT	367	Median, 33 (IQR, 25-50)	61.0	241	49	Consensus on Concussion in Sport	≥3 Symptoms, ≥2 severity on RPQ
Zuckerman et al,^23^ 2016	US	Sport (NCAA, ISP)	Prospective cohort	1507	NA	31.2	1507	112	Consensus on Concussion in Sport	≥1 Symptom
**3 Months**										
Bazarian et al,^25^ 1999	US	ED	Prospective case-control	71	Mean, 29.0	49.3	69	30	GCS 15, LOC <10 min, no abnormal CT findings	*DSM-IV*
Dischinger et al,^29^ 2009	US	ED	Prospective cohort	180	Mean, 35	36.1	110	46	ACRM 1993	≥4 Symptoms on concussion symptom checklist
Faux et al,^30^ 2011	Australia and Canada	ED	Prospective cross-validation	207	Australia: mean, 33.6; Canada: mean, 37.9	Australia, 22.0; Canada, 34.5	155	47	ACRM 1993	≥3 Symptoms, ≥2 on RPQ
Hou et al,^31^ 2012	England	ED	Prospective cohort	126	Mean (SD), 38.3 (14.1)	37.3	108	24	ACRM 1993	≥3 Symptoms on RPQ
Ponsford et al,^27^ 2012	Australia	ED	Prospective case-control	123	Median, 31 (range, 18-72)	26.0	90	41	LOC <30 min, PTA <24 h, GCS 13-15	≥1 Symptom on PCSS
Rowe et al,^36^ 2021	Canada	ED	Prospective cohort	250	Median, 35 (IQR, 23-49)	52.4	183	91	WHO	≥1 Symptom at ≥2 severity on RPQ
Sheedy et al,^32^ 2009	Australia	ED	Prospective cohort	100	Mean (SD), 33.6 (12.7)	22.0	78	20	ACRM 1993	≥3 Symptoms on RPQ
**6 Months**										
Caplain et al,^28^ 2017	France	ED	Prospective cohort	86	Mean (SD), 34.8 (11.3)	30.6	72	23	ACRM 1993	*DSM-IV*
Hou et al,^31^ 2012	England	ED	Prospective cohort	126	Mean (SD), 38.3 (14.1)	37.3	108	22	ACRM 1993	≥3 Symptoms on RPQ
Langer et al,^24^ 2021	Canada	Health record (OHIP, NACRS)	Retrospective medical record review	587 057	Range, 18-60	42.2	587 057	73 122	*ICD-9* and *ICD-10* codes	≥2 Visits for symptoms

Abbreviations: ACRM, American Congress of Rehabilitation Medicine; CT, computed tomography; *DSM-IV*, *Diagnostic and Statistical Manual of Mental Disorders (Fourth Edition)*; ED, emergency department; EMED, Expeditionary Medical Encounter Database; GCS, Glasgow Coma Scale; *ICD-9*, *International Classification of Diseases, Ninth Revision*; *ICD-10*, *International Statistical Classification of Diseases and Related Health Problems, Tenth Revision*; ISP, Injury Surveillance Program; LOC, loss of consciousness; mTBI, mild traumatic brain injury; NA, not applicable; NACRS, National Ambulatory Care Reporting System; NCAA, National Collegiate Athletic Association; OHIP, Ontario Health Insurance Plan; PCSC, Post-Concussion Symptom Checklist; PSAC, persisting symptoms after concussion; PTA, posttraumatic amnesia; RCT, randomized clinical trial; RPQ, Rivermead Post-Concussion Symptoms Questionnaire; WHO, World Health Organization.

### Characteristics of Included Studies

In total, 597 877 participants were included in the 34 studies.^19,20,21,22,23,24,25,26,27,28,29,30,31,32,33,34,35,36,37,38,39,40,41,42,43,44,45,46,47,48,49,50,51,52^ Timing of initial variable collection ranged from 2 hours to 21 days after concussion. In 3 of the 34 studies (8.8%) performing retrospective medical record reviews, 1 (33.3%) recorded sports-related concussion in real time on an electronic tracking program,^23^ while 2 (66.7%) did not specify timing of acutely collected initial variables.^24,50^

Various definitions were used to diagnose mTBI or concussion; most commonly, studies used the Glasgow Coma Scale (score of 13-15 on a scale of 3-15, with 3 indicating most severe impairment), posttraumatic amnesia (<24 hours), loss of consciousness (LOC; <30 minutes), and no abnormal intracranial findings.^21,22,25,26,27,39,40,41,42,49,52^ Other definitions used included the American Congress of Rehabilitation Medicine 1993 definition^28,29,30,31,32,43,44,48,53^; the Department of Veterans Affairs and US Department of Defense guidelines^20,38,44,51,54^; the Consensus on Concussion in Sport definition^23,33,46,47,55^; *International Classification of Diseases, Ninth Revision (ICD-9)* codes 850.0, 850.1, 850.11, 850.12, 850.5, or 850.9^24,34,50,56^; the World Health Organization definition^35,36,57^; the Zurich Consensus Statement^37,58^; and physician diagnosis.^19^

The 15 studies included in the meta-analysis enrolled a total of 592 406 participants with mean age of 29.3 years (range, 16-89 years) (42.2% female, 57.8% male) with no intracranial abnormalities.^23,24,25,26,27,28,29,30,31,32,33,34,35,36,37^ The primary outcome time points were 1 month,^23,26,33,34,35,37^ 3 months,^27,29,30,32,36^ and 6 months^24,28^ after concussion (Table). One study examined PSAC at 1 and 3 months,^25^ and another examined PSAC at 3 and 6 months after concussion.^31^ The most common definition of PSAC was 1 or more symptoms reported on the Post-Concussion Symptom Checklist (PCSC)^23,27,33^ or the Rivermead Post-Concussion Symptoms Questionnaire (RPQ),^35,36^ followed by 3 or more symptoms on the RPQ^30,31,32,37^; *Diagnostic and Statistical Manual of Mental Disorders, Fourth Edition (DSM-IV)* codes^25,28^; *ICD-9* codes^34^; *International Statistical Classification of Diseases and Related Health Problems, Tenth Revision* criteria^26,59^; 4 or more symptoms on the PCSC^29^; and 2 or more physician visits for concussion symptoms^24^ (Table). A total of 733 of 4121 participants (17.8%) met criteria for PSAC at 1 month, 299 of 793 (37.7%) met criteria at 3 months, and 73 167 of 587 237 (12.5%) met criteria at 6 months (Table).

The most common recruitment location for the studies included in the meta-analysis was emergency departments (EDs) (11 of 15 studies [73.3%]).^25,26,27,28,29,30,31,32,35,36,37^ The 4 other studies (26.7%) recruited participants from sports settings,^23^ military settings,^34^ a specialized concussion clinic,^33^ and primary care physicians.^24^ The recruitment settings for studies that examined PSAC at 1 month were mainly EDs^25,26,35,37^ but also military settings,^34^ sports settings,^23^ and a specialized concussion clinic.^33^ Studies examining PSAC at 3 months were exclusively EDs,^25,27,29,30,31,32,36^ and those examining PSAC at 6 months were EDs^28,31^ or primary care physicians.^24^

### Risk of Bias

Fifteen of the 34 included studies (44.1%) were assessed as having high risk of bias, 10 (29.4%) as having moderate risk, and 9 (26.5%) as having low risk. High risk of bias was mainly attributed to high attrition or lack of reporting on loss to follow-up (eTable 4 in Supplement 1).

### Factors Associated With PSAC by Time Point After Concussion

**Figure 1.**
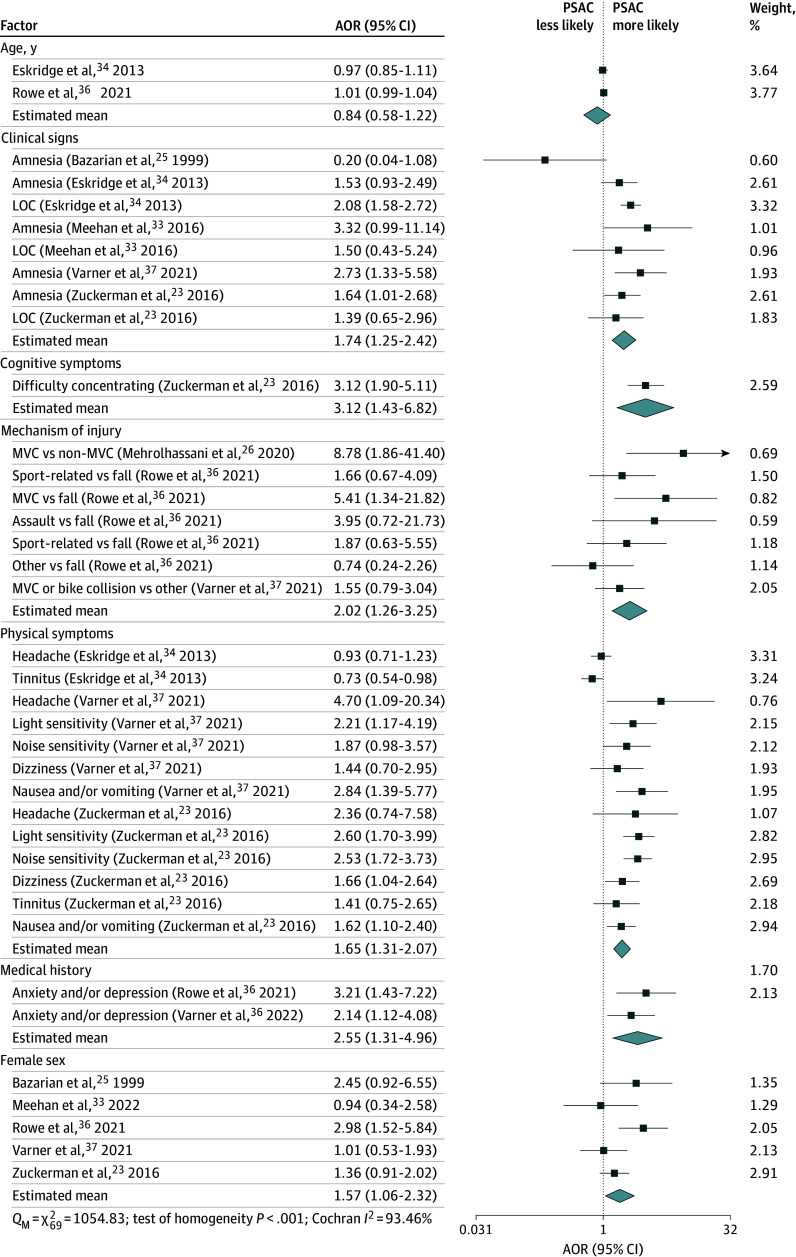
Meta-Analysis of Factors Associated With Persisting Symptoms After Concussion (PSAC) at 1 Month Weights are from the unadjusted random-effects model. Group means are from the adjusted multilevel model. Higgins-Thompson proportion of variance from between-studies heterogeneity, *I*^2^ = 79.24%; Higgins-Thompson proportion of variance from between–risk factor heterogeneity, *I*^2^ = 16.96%. Data for additional categories are given in eFigure 2 in Supplement 1. AOR indicates adjusted odds ratio; LOC, loss of consciousness; MVC, motor vehicle collision; *Q*_M_, multilevel Cochran residual homogeneity test statistic.

The factor associated with greatest odds of PSAC at 1 month was cognitive symptoms, specifically difficulty concentrating (AOR, 3.12 [95% CI, 1.43-6.82]). This was followed by medical history of anxiety and/or depression (AOR, 2.55 [95% CI, 1.31-4.96]) and mechanism of injury, specifically motor vehicle collisions (AOR, 2.02 [95% CI, 1.26-3.25]) (Figure 1 and eFigure 2 in Supplement 1).

**Figure 2.**
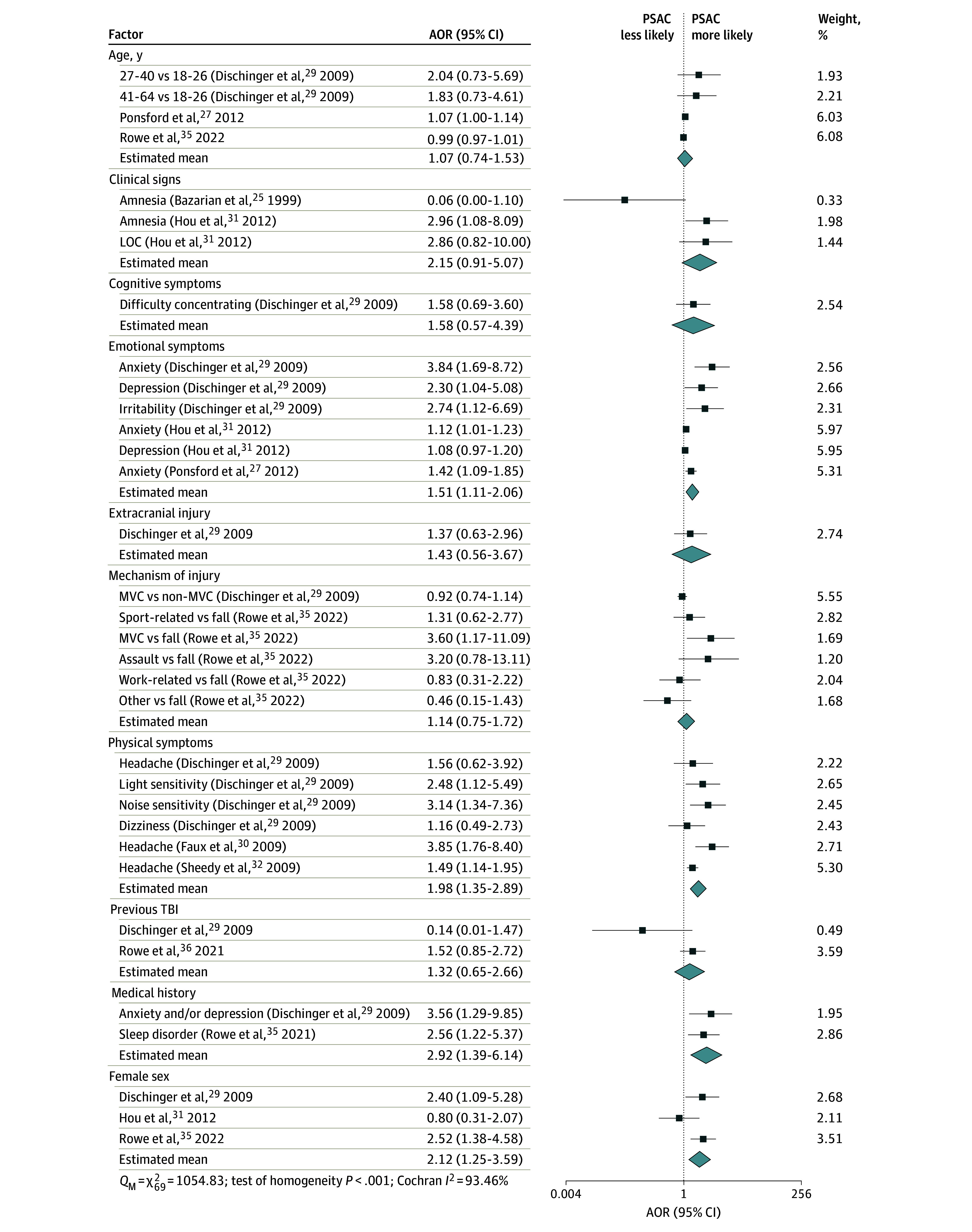
Meta-Analysis of Factors Associated With Persisting Symptoms After Concussion (PSAC) at 3 Months Weights are from the unadjusted random-effects model. Group means are from the adjusted multilevel model. Higgins-Thompson proportion of variance from between-studies heterogeneity, *I*^2^ = 79.24%; Higgins-Thompson proportion of variance from between–risk factor heterogeneity, *I*^2^ = 16.96%. AOR indicates adjusted odds ratio; LOC, loss of consciousness; MVC, motor vehicle collision; *Q*_M_, multilevel Cochran residual homogeneity test statistic; TBI, traumatic brain injury.

At 3 months, the factor associated with greatest odds of PSAC was medical history of anxiety and/or depression or of sleep disorders (AOR, 2.92 [95% CI, 1.39-6.14]). This was followed by female sex (AOR, 2.12 [95% CI, 1.25-3.59]) and physical symptoms, including headache, dizziness, and light and noise sensitivity (AOR, 1.98 [95% CI, 1.35-2.89]) (Figure 2). Of note, although not significantly associated with PSAC, clinical signs, including amnesia and LOC, may be of clinical relevance based on the point estimate (AOR, 2.15 [95% CI, 0.91-5.07]).

**Figure 3.**
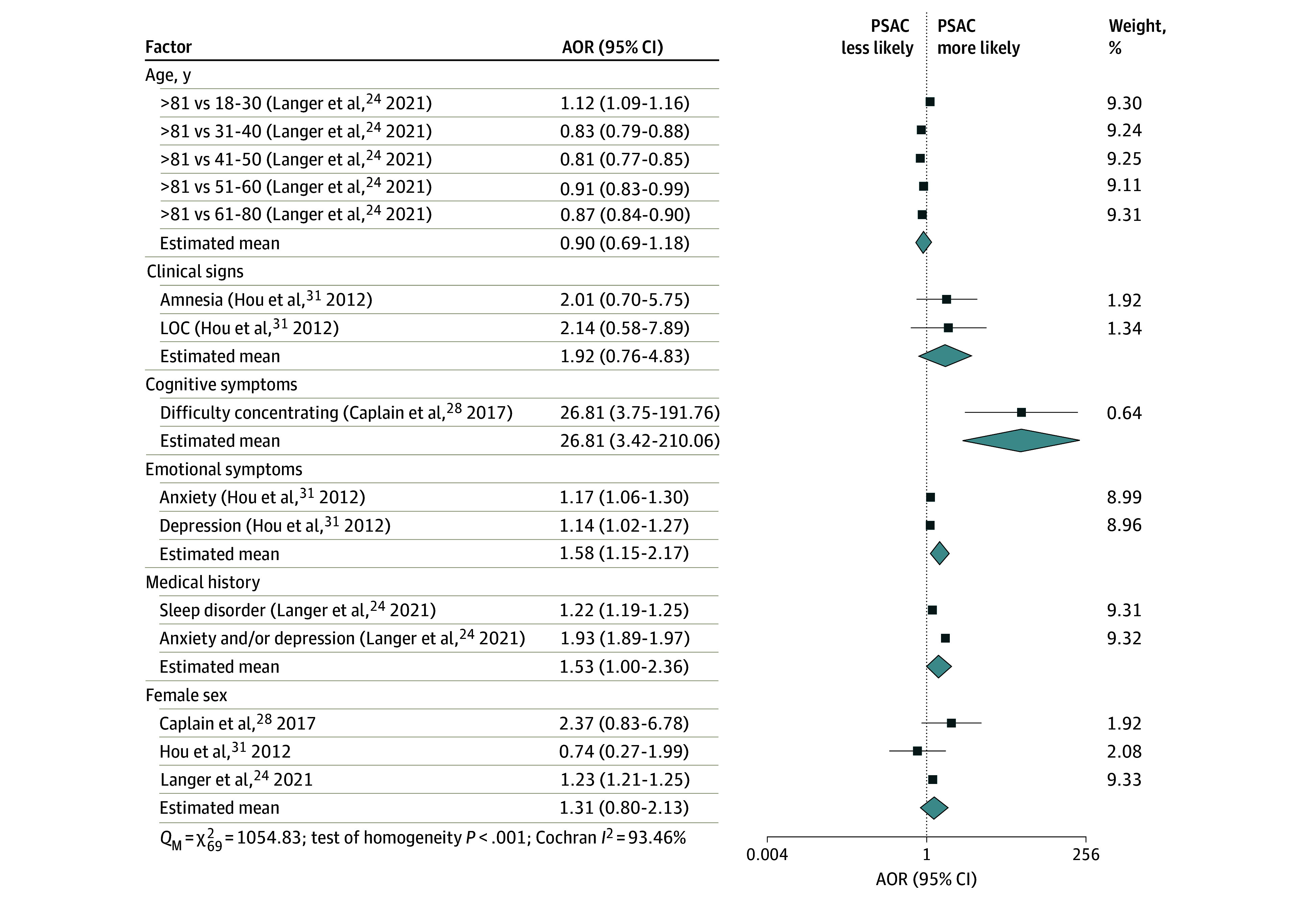
Meta-Analysis of Factors Associated With Persisting Symptoms After Concussion (PSAC) at 6 Months Weights are from the unadjusted random-effects model. Group means are from the adjusted multilevel model. Higgins-Thompson proportion of variance from between-studies heterogeneity, *I*^2^ = 79.24%; Higgins-Thompson proportion of variance from between-risk-factor heterogeneity, *I*^2^ = 16.96%. AOR indicates adjusted odds ratio; LOC, loss of consciousness; *Q*_M_, multilevel Cochran residual homogeneity test statistic.

The factor associated with greatest odds of PSAC at 6 months was cognitive symptoms (difficulty concentrating; AOR, 26.81 [95% CI, 3.42-210.06]). This was followed by emotional symptoms (AOR, 1.58 [95% CI, 1.15-2.17]) and medical history of anxiety and/or depression or of sleep disorders (AOR, 1.53 [95% CI, 1.00-2.36]) (Figure 3). Clinical signs may also be of clinical relevance based on the point estimate (AOR, 1.92 [95% CI, 0.76-4.83]) but was not statistically significant.

Overall, high heterogeneity was observed across studies (Cochran *I*^2^ = 93.46%), with evidence of heterogeneity driving some results (Cochran *Q* statistic = χ^2^_69_ = 1054.83; *P* < .001). The Higgins-Thompson *I^2^* statistics showed that this was largely due to between-study heterogeneity (*I*^2^ = 79.24%) and less so between-factor heterogeneity (*I*^2^ = 16.96%).

### Factors Associated With PSAC at All Time Points After Concussion

**Figure 4.**
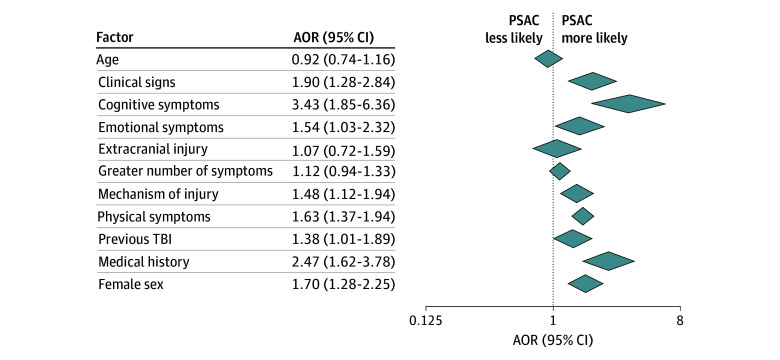
Meta-Analysis of Factors Associated With Persisting Symptoms After Concussion (PSAC) Across All Time Points Adjusted odds ratios (AORs) with corresponding 95% CIs for average marginal effect estimates from the adjusted multilevel model are shown. Predefined categories included clinical signs (loss of consciousness, amnesia), cognitive symptoms (difficulty concentrating), emotional symptoms (acute anxiety and/or depression, irritability), mechanism of injury (motor vehicle collision, fall, assault, work-related, or sport-related), physical symptoms (headache, light sensitivity, noise sensitivity, dizziness, nausea and/or vomiting, or tinnitus), and medical history (anxiety and/or depression, sleep disorders). TBI indicates traumatic brain injury.

Across all time points, the factor associated with greatest odds of PSAC was cognitive symptoms (difficulty concentrating; AOR, 3.43 [95% CI, 1.85-6.36]) followed by medical history of anxiety and/or depression or of sleep disorders (AOR, 2.47 [95% CI, 1.62-3.78]) and clinical signs (amnesia and LOC; AOR, 1.90 [95% CI, 1.28-2.84]) (Figure 4). Female sex was also associated with increased odds of PSAC across all time points (AOR, 1.70 [95% CI, 1.28-2.25]). Although the factors associated with greatest odds of PSAC varied by time after concussion, visual inspection of the overall effect estimates for common variables across time points suggests that associations for several factors remained consistent in direction and had similar ORs, suggesting no modification of associations.

## Discussion

In this systematic review and meta-analysis, we evaluated factors associated with PSAC in adults more than 1 month after concussion. Due to heterogeneity in defining PSAC, we stratified our analysis by time after injury to capture unique factors associated with PSAC at 1, 3, and 6 months. We found that cognitive symptoms (difficulty concentrating) were associated with greatest odds of PSAC at 1 and 6 months, while medical history of anxiety and/or depression or of sleep disorders was associated with greatest odds of PSAC at 3 months. Across all time points, cognitive symptoms, medical history, and clinical signs were associated with greatest odds of PSAC. Other variables, including age, extracranial injury, and previous TBI, were relatively less important.

Two prior systematic reviews examining multivariable prognostic models^10^ and risk factors of PSAC in adults^11^ included individuals with intracranial abnormalities and pediatric patients and were limited by primary recruitment from EDs. A recent overview of systematic reviews evaluated factors associated with PSAC beyond 3 months; however, methodologic design was limited by assessing secondary research, and the study failed to identify conclusive risk factors of PSAC.^13^ The present meta-analysis advances these findings by including primary studies examining factors associated with PSAC in adults without evidence of intracranial injury, as this distinction permits these factors to be representative of injury severity specific to the adult population with concussion.

In our analysis, cognitive symptoms, medical history, and mechanism of injury were associated with the greatest odds of PSAC in adults 1 month after concussion. Individuals who reported difficulty concentrating acutely after concussion were 3 times more likely to develop PSAC. However, the causality of concentration difficulties cannot be ascertained due to the multifactorial nature of concussion symptoms. Physical symptoms, disturbed sleep, and pain may contribute to difficulty concentrating. Previous network analysis of PSAC identified concentration difficulties as a central symptom at both 6 weeks and 10 years after concussion.^60^ These findings and those presented herein may suggest that difficulty concentrating has an important role acutely after concussion in the development of PSAC.

Medical history of anxiety and/or depression was identified as the factor associated with the second greatest odds of PSAC at 1 month after concussion and across all time points. Previous findings support these results, identifying medical history of psychiatric disorders and mental health before injury as commonly reported risk factors for PSAC in adults.^11,13^

Mechanism of injury, specifically motor vehicle collisions compared to other mechanisms, was associated with increased odds of PSAC at 1 month. Although previous reviews have not found mechanism of injury to be a significant risk factor,^10,11,13^ our results suggest that injury mechanism may be relevant for PSAC at 1 month after concussion only.

The factor associated with greatest odds of PSAC at 3 months after concussion was medical history of anxiety and/or depression or of sleep disorders. Adults with these comorbidities were approximately 3 times more likely to develop PSAC than those without. One study included in our systematic review determined that pharmacologic treatment for depression at the time of concussion was associated with significantly fewer clinician visits for concussion symptoms at 3, 6, and 12 months after injury, highlighting the importance of mental health screening and treatment concurrent with concussion care.^50^

Female sex emerged as a factor associated with PSAC at 3 months. Female sex was also associated with increased odds of PSAC across all time points in our analysis. Our results align with a previous review which reported that female sex was a consistent independent factor associated with worse outcomes after mTBI.^10^

Acute physical symptoms, including headache, tinnitus, light sensitivity, noise sensitivity, dizziness, and nausea and/or vomiting, were associated with increased risk of PSAC at 3 months after concussion by an AOR of 1.98. Headache and neck pain occurring acutely after concussion have been highlighted as factors associated with PSAC.^11^ Nausea and/or vomiting have also been reported to be important factors associated with PSAC.^13^ Though Silverberg et al^10^ reported initial symptom severity may be associated with PSAC, our findings suggest that the presence of specific symptoms experienced acutely after concussion, such as difficulty concentrating, headache, or nausea and/or vomiting, may be more important to evaluate than the sheer number of symptoms when evaluating concussion prognosis.

At 6 months after concussion, the factors associated with greatest odds of PSAC in our analysis were cognitive symptoms, emotional symptoms, and medical history of anxiety and/or depression or of sleep disorders. Cognitive symptoms (difficulty concentrating) were associated with greatest odds of PSAC at 6 months after concussion and may therefore have an influential prognostic role across several time points after concussion.

In addition, acute emotional symptoms (anxiety and depressive symptoms) appeared as factors associated with PSAC at 6 months. Acute anxiety was also identified to have an association with poor outcome by Silverberg et al.^10^ Though distinct from medical history of depression and/or anxiety, which was associated with PSAC at 1 and 3 months, these findings highlight that both premorbid and acute anxiety and/or depression are important for estimating PSAC.

The factor associated with greatest odds of PSAC across all time points was cognitive symptoms, specifically difficulty concentrating. Although other cognitive symptoms, such as slowness to respond and difficulty remembering, were not examined in the studies in this review, these cognitive symptoms have been reported as significant predictors of PSAC in a pediatric population.^61^ Our findings provide a basis to support the evaluation and monitoring of cognitive symptoms in acute care settings, as they may be associated with PSAC in adults.

Medical history of anxiety and/or depression or of sleep disorders was associated with second highest odds of PSAC. Our findings and those previously^11,13^ highlight an association between preinjury medical history of mental health and sleep disorders in adults and the risk of developing PSAC. Monitoring and treatment of these comorbid conditions should be considered throughout recovery. Clinical signs, including LOC and amnesia, were associated with the third greatest odds of PSAC across all time points. These variables may act as relative markers of injury severity whereby the presence of LOC and/or amnesia indicate more severe injury. Prior literature remains divided on the significance of clinical signs in predicting outcome; 1 study in our analysis found amnesia to be protective,^25^ while another suggested LOC was associated with greatest risk of PSAC.^34^ Our analysis suggests that these variables may be markers of injury severity and may be important risk factors for concussion recovery.

### Limitations

This study has limitations. There was high between-study heterogeneity in this meta-analysis. This was expected due to variability and lack of consensus in diagnostic criteria for PSAC, specifically timing of PSAC evaluation and threshold for symptom severity. Heterogeneity in timing of evaluation resulted in different studies being evaluated or excluded across time points. At 1 month after concussion, 17.8% of participants met criteria for PSAC; at 3 months, 37.7%; and at 6 months, 12.5%, as defined by independent studies. Additionally, lack of consensus regarding the symptom severity threshold to define PSAC likely contributed to this heterogeneity. The most common threshold for PSAC was 1 or more symptoms on the PCSC, which likely overestimated the prevalence of PSAC due to the nonspecific nature of concussion symptoms. Recruitment setting may have also influenced the prevalence of PSAC; studies evaluating 1-month outcomes were the most diverse in terms of recruitment location, including EDs,^25,26,35,37^ military settings,^34^ sports settings,^23^ and a specialized concussion clinic.^33^ Conversely, all studies examining outcomes at 3 months recruited from EDs exclusively,^25,27,29,30,31,32,36^ and studies examining 6-month outcomes were recruited from EDs^28,31^ or primary care physicians.^24^

Although age was commonly investigated as a potential risk factor for PSAC, older age did not emerge as a factor associated with PSAC in this meta-analysis. Inconsistent reporting of age across studies contributed to the high heterogeneity observed in our meta-analysis and added to the difficulty of assessing age as a risk factor for PSAC in previous literature.^11,62^ Though Lubbers et al^11^ hypothesized older age would be a significant risk factor for PSAC, most of their included studies assessed age as a continuous variable, complicating the calculation of effect sizes for specific age groups. However, when age was expressed as both a continuous and a categorical variable, Le Sage et al^62^ reported an association with PSAC only when age was treated categorically. Notably, of the 6 studies examining age in the current meta-analysis,^24,27,29,34,35,36^ 4 reported age as a continuous variable^27,34,35,36^; therefore, an analysis of adult subgroups may be necessary to analyze the association between age and PSAC.

Visual examination revealed little evidence of modification of the overall effect estimates for common factors associated with PSAC across the time points. This remains an opportunity for future evaluation. In addition, the results of our analysis are based on a restricted sample of 592 406 adults with no intracranial abnormalities and therefore may show conflicting results with previous literature with broader inclusion criteria.

## Conclusions

This systematic review and meta-analysis found the factors associated with greatest odds of PSAC were acute cognitive symptoms (concentration difficulties), medical history (anxiety and/or depression, sleep disorders), and clinical signs (LOC, amnesia) across all included time points after concussion. Future longitudinal studies should consider evaluating acute risk factors of PSAC at different time points after concussion in addition to evaluating how risk factors may be combined to create a multivariable prediction model.

## Notes

Supplement 1. **eTable 1.** Example Search Strategy **eTable 2.** Eligibility Criteria **eFigure 1.** Study Flow Diagram **eTable 3.** Characteristics of Studies Included in Systematic Review **eTable 4.** QUIPs Risk of Bias Assessment **eFigure 2.** Meta-Analysis of Factors Associated With Persisting Symptoms After Concussion at 1 Month

Supplement 2.
Data Sharing Statement
