# Supplementary material for: Factors Associated With Persisting Symptoms After Concussion in Adults With Mild TBI: A Systematic Review and Meta-Analysis
Source: JAMA Netw Open. 2025 Jun 18;8(6):e2516619. doi: 10.1001/jamanetworkopen.2025.16619 (PMC12177663; doi:10.1001/jamanetworkopen.2025.16619)
Supplement: Supplement 1. — eTable 1. Example Search Strategy eTable 2. Eligibility Criteria eFigure 1. Study Flow Diagram eTable 3. Characteristics of Studies Included in Systematic Review eTable 4. QUIPs Risk of Bias Assessment eFigure 2. Meta-Analysis of Factors Associated With Persisting Symptoms After Concussion at 1 Month [file jamanetwopen-e2516619-s001.pdf]

Supplementary Online Content

McIntosh SJ, Vergeer MH, Galarneau JM, Eliason PH, Debert CT. Factors associated with persisting symptoms after concussion in adults with mild TBI: a systematic review and meta-analysis. *JAMA Netw Open*. 2025;8(6):e2516619.
doi:10.1001/jamanetworkopen.2025.16619

eTable 1. Example Search Strategy
eTable 2. Eligibility Criteria
eFigure 1. Study Flow Diagram
eTable 3. Characteristics of Studies Included in Systematic Review
eTable 4. QUIPs Risk of Bias Assessment
eFigure 2. Meta-Analysis of Factors Associated With Persisting Symptoms After Concussion (PSAC) at 1 Month

This supplementary material has been provided by the authors to give readers additional information about their work.

© 2025 McIntosh SJ et al. *JAMA Network Open*.

eTable 1. Example Search Strategy

Embase search strategy
1. exp brain concussion/
2. concuss*.tw,kf.
3. mild traumatic brain injur*.tw,kf.
4. mTBI.tw,kf.
5. SRC.tw,kf.
6. traumatic brain injury.tw,kf.
7. TBI.tw,kf.
8. concussional syndrome*.tw,kf.
9. sports related concussion.tw,kf.
10. ((mild or minimal or minor) adj2 head injur*).tw,kf.
11. ((brain or cerebral or craniocerebral or cranio-cerebral or intra-cranial or intracranial) adj2 (concuss* or hematoma* or haematoma* or injur* or contus*)).tw,kf.
12. 1 or 2 or 3 or 4 or 5 or 6 or 7 or 8 or 9 or 10 or 11
13. clinical measure*.tw,kf.
14. (symptom* adj2 report*).tw,kf.
15. (injur* adj2 characteristic*).tw,kf.
16. demographic*.tw,kf.
17. previous medical histor*.tw,kf.
18. predict*.tw,kf.
19. 13 or 14 or 15 or 16 or 17 or 18
20. exp post-concussion syndrome/
21. persistent post-concussive syndrome.tw,kf.
22. persistent post-concussive symptoms.tw,kf.
23. PPCS.tw,kf.
24. PCS.tw,kf.
25. exp Glasgow Coma Scale/ or exp Glasgow Outcome Scale/
26. extended glasgow coma outcome scale.tw,kf.
27. Glasgow coma scale.tw,kf.
28. Glasgow outcome scale.tw,kf.
29. Rivermead post-concussion questionnaire.tw,kf.
30. length of time to recovery.tw,kf.
31. time to recovery.tw,kf.
32. length to return to sport.tw,kf.
33. length to return to play.tw,kf.
34. return to sport.tw,kf.
35. return to play.tw,kf.
36. 20 or 21 or 22 or 23 or 24 or 25 or 26 or 27 or 28 or 29 or 30 or 31 or 32 or 33 or 34 or 35
37. exp prognosis/
38. prognos*.tw,kf.
39. 37 or 38
40. 12 and 19 and 36 and 39
41. case series.tw,kf.
42. case report.tw,kf.
43. systematic.mp. or review.ti. [mp=title, abstract, heading word, drug trade name, original title, device manufacturer, drug manufacturer, device trade name, keyword heading word, floating subheading word, candidate term word]
44. meta analysis.ti.
45. opinion.ti.
46. editorial.ti.
47. 41 or 42 or 43 or 44 or 45 or 46

© 2025 McIntosh SJ et al. *JAMA Network Open*.

48. animal.tw,kf.
49. rat.tw,kf.
50. rats.tw,kf.
51. mice.tw,kf.
52. primate*.tw,kf.
53. rabbit*.tw,kf.
54. mouse.tw,kf.
55. 48 or 49 or 50 or 51 or 52 or 53 or 54
56. 40 not 47
57. 56 not 55
58. limit 57 to yr="1970 -Current"
59. limit 58 to ("all adult (19 plus years)" or "adolescent (13 to 18 years)" or "young adult (19 to 24 years)" or "adult (19 to 44 years)" or "young adult and adult (19-24 and 19-44)" or "middle age (45 to 64 years)" or "middle aged (45 plus years)")

© 2025 McIntosh SJ et al. *JAMA Network Open*.

eTable 2. Eligibility Criteria

Eligibility Criteria	Inclusion	Exclusion
Participants	Clinician diagnosed mild traumatic brain injury (mTBI) or concussion group with mean age ≥18 and ≤65 years	Pediatric participants (mean age <18 years), older adults (mean age >65 years), Glasgow Coma Scale (GCS) <13, moderate-severe traumatic brain injury without a mTBI sub-group, self-reported mTBI or concussion without clinician diagnosis, imaging (CT, MRI) as the primary risk factor, positive diagnostic imaging findings
Measures	Any risk factors collected ≤1 month post-injury	Risk factors collected >1 month post-injury
Comparators	If available, a control group without acute mTBI	
Outcome	Any negative outcome ≥1-month post-injury including persisting post-concussion symptoms (PPCS), persisting symptoms after concussion (PSaC), specific symptom severity ratings (Rivermead post-concussion symptom questionnaire [RPQ], post-concussion symptom checklist [PCSC]), disability measured with the Glasgow Outcome Scale Extended (GOSE), post-traumatic stress disorder, prolonged return to work/school or play/sport, length of time to recovery	Outcome collected <1-month post-injury
Study Type	Studies with a longitudinal design including original research manuscripts published in peer-reviewed journals including cohort studies, randomized control trials, case control trials, and cross-sectional studies	Gray literature and original research including case series, single case reports, reviews, expert opinions, pre-clinical studies, meta-analysis, and systematic reviews, randomized control trials were excluded if they did not include a natural recovery group with no intervention

© 2025 McIntosh SJ et al. *JAMA Network Open*.

eFigure 1. Study Flow Diagram

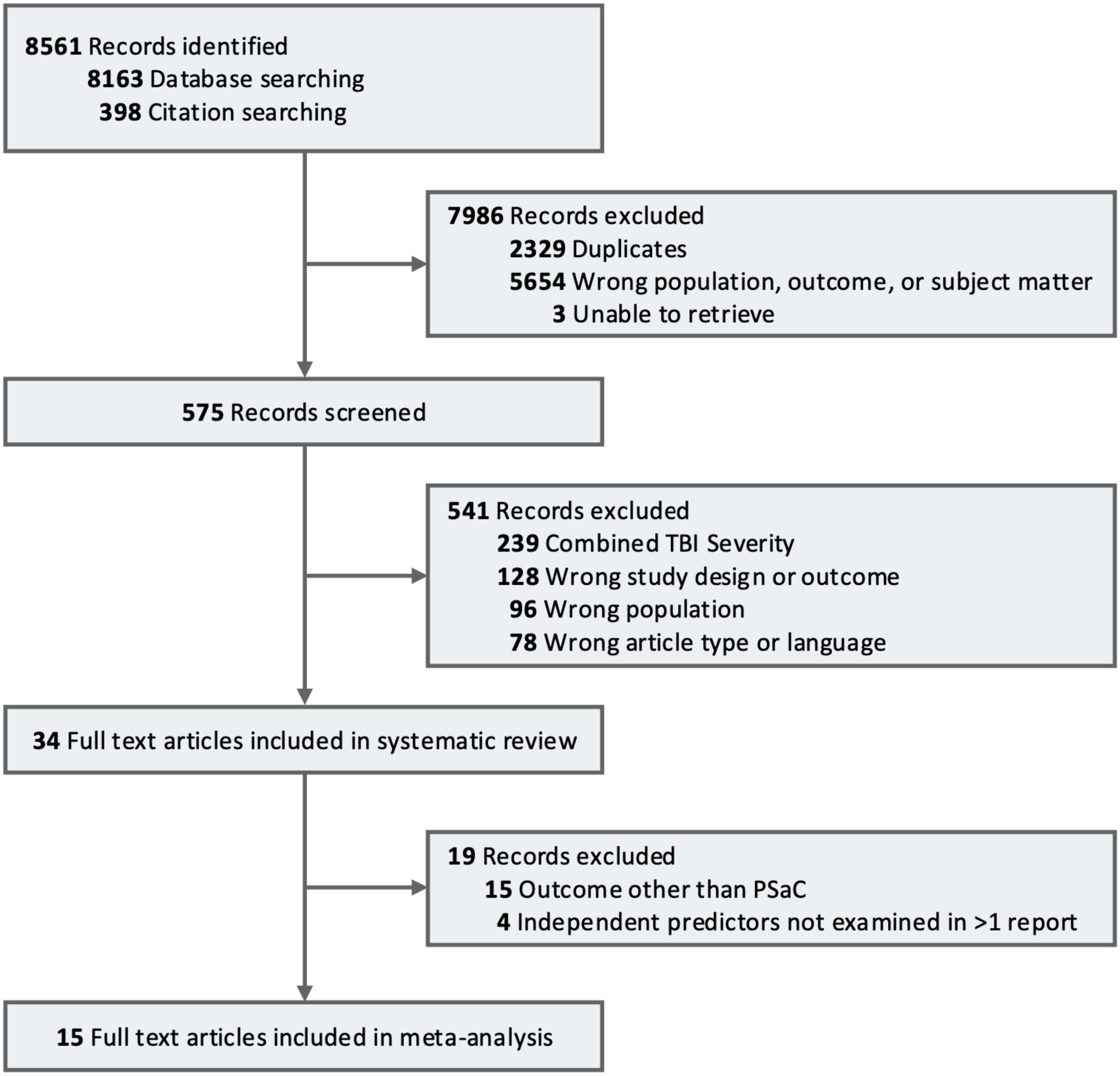

Abbreviations: PSaC, Persisting symptoms after concussion; TBI, traumatic brain injury.

© 2025 McIntosh SJ et al. *JAMA Network Open*.

eTable 3. Characteristics of Studies Included in Systematic Review

Source	Country	Setting	Design	Sample	Age, (% female)	mTBI diagnostic criteria	Mechanism of injury	Timepoints (number of participants)	Predictor variables [estimate (95% CI)]
**Persisting symptoms after concussion**									
Bazarian et al,[25] 1999	United States	ED	Prospective case-control	N=83[a], n=71[b]	29.0, (49.3%)	GCS 15, LOC <10min, CT-	MVC (39.4%), MCC (1.4%), bicycle (4.2%, pedestrian struck (2.8%), fall (16.9%), sport (21.1%), assault (1.4%), projectile (5.6%), other (7.0%)	Initial (<24 hrs): (n=71)<br>Follow-up:<br>• 1 month (n=69)<br>• 3 months (n=69)<br>• 6 months (n=65)	<u>1 month</u>: Retrograde and anterograde amnesia[d] [OR=0.055 (0.002, 0.465)], female[d] [OR=7.87 (1.98, 41.67)], digit span test[d] [OR=0.748 (0.521, 1.032)], Hopkins verbal learning A[d] [OR=0.786 (0.654, 0.914)]<br><u>3 months</u>: Retrograde and anterograde amnesia[d] [OR=0.130 (0.0, 0.935)], digit span test[d] [OR=0.744 (0.576, 0.936)]
Caplain et al,[28] 2017	France	ED	Prospective cohort	N=86[a], n=72[b]	34.8±11.3, (30.6%)	ACRM 1993	Attack (27.8%), fall (18.1%), workplace injury (12.5%), road accident (18.1%), other (12.5%)	Initial (8-21 days): (n=86)<br>Follow-up (6 months): (n=72)	<u>6 months</u>: Difficulty concentrating[d] [OR=26.81 (3.75, 191.85)], global QoL[d] [OR=9.56 (1.36, 67.06)], verbal phonemic fluency[d] [OR=12.33 (1.60, 95.03)]
Dischinger et al,[29] 2009	United States	ED	Prospective cohort	N=180[a,b]	35, (36%)	ACRM 1993	MVC (55%)	Initial (<3 days): (n=180)<br>Follow-up:<br>• 3-10 days (n=164)<br>• 3 months (n=110)	<u>3 months</u>: Noise sensitivity[d] [OR=3.06 (1.09, 9.04)], anxiety (+)/female[d] [OR=10.15 (2.68, 38.54)], anxiety (-)/female[d] [OR=0.18 (0.03, 1.01)], female/anxiety (+)[d] [OR=48.66 (7.50, 315.8)], male/anxiety (+)[d] [OR=0.87 (0.29, 2.54)]
Eskridge et al,[34] 2013	United States	Military (EMED)	Retrospective chart review	N=1713[a], n=1656[b]	24.1±4.9 years, (0%)	ICD-9 codes	Combat/blast-related (100%)	Initial (<48 hrs): (n=1666)<br>Follow-up (>1 month): (n=1656)	<u>1 month</u>: Age [OR=1.01 (0.88, 1.16)], extracranial injury [OR=1.00 (0.99, 1.00)], LOC[d] [OR=2.08 (1.56, 2.77)], altered mental status[d] [OR=1.53 (1.07, 2.17)], amnesia [OR=1.29 (0.78, 2.15)], headache [OR=1.11 (0.81, 1.50)], tinnitus [OR=0.73 (0.54, 1.00)], previous blast[d] [OR = 1.83 (1.15, 2.90)], previous concussion [OR=1.15 (0.64, 2.07)]

© 2025 McIntosh SJ et al. *JAMA Network Open*.

Faux et al,[30] 2011	Australia and Canada	ED	Prospective cross-validation	N = 207[a,b]	Australia: 33.6, (22%); Canada: 37.9, (35%)	ACRM 1993	Australia: assault (40%), MVC (13%), fall (24%), collision with object or other (23%); Canada: assault (19%), MVC (43%), fall (31%), collision with object or other (7%)	Initial (<24 hrs): (n=207) Follow-up (3 months): (n=155)	3 months: Canada: Immediate verbal recall[d] [OR=0.704 (0.516, 0.960)], delayed verbal recall [OR=1.292 (0.897, 1.861)], headache[d] [OR=3.689 (1.249, 10.898); Combined (Canada & Australia): Immediate verbal recall[d] [OR=0.661 (0.515, 0.848)], delayed verbal recall [OR=0.916 (0.681, 1.231)], headache[d] [OR=3.850 (1.764, 8.401), blood alcohol level[d] [OR=0.268 (0.085, 0.848)]
Foster et al,[19] 2022	Canada	mTBI clinic	Prospective cohort	N=209 [a], n=167 [b]	32.9±12.2, (59%)	Physician diagnosed	Fall (32%), exercise/sport (29%), transportation (22%), violence (9%), direct head impact (8%)	Initial (<7 days): (n=167) Follow-up (4-16 weeks): (n=81)	8 weeks: UPSIT (Smokers included) [OR=1.05 (0.91–1.20)], UPSIT (Smokers excluded) [OR=1.00 (0.88, 1.14)]
Hou et al,[31] 2012	England	ED	Prospective cohort	N=126[a], n=107[b]	38.3±14.1, (37%)	ACRM 1993	Not reported	Initial (<2 weeks): (n=126) Follow-up: • 3 months (n=107) • 6 months (n=107)	3 months: BRIQ (all-or-nothing)[d] [OR=1.141 (1.050, 1.240)] 6 months: BRIQ (all-or-nothing) [OR=1.106 (0.991, 1.233)], BIPQ (illness identity)[d] [OR=1.053 (1.008, 1.101)]
Langer et al,[24] 2021	Canada	Health Records (OHIP & NACRS)	Retrospective chart review	N=587 057[a], n=430 602[c]	18-60, (42.2%)	ICD-9 and ICD-10 codes	Not reported	Initial (unspecified): (n=430 602) Follow-up (>6 months): (n=430 602)	6 months: Bipolar disorder[d] [OR=4.28 (3.86, 4.76)], personality disorder[d] [OR=2.40 (2.31, 2.50)], anxiety/depression[d] [OR=1.32 (1.31, 1.34)], other mental health disorder[d] [OR=1.34 (1.31, 1.37)], >15 primary care visits[d] [OR=5.51 (5.41, 5.62)], 8-14 primary care visits[d] [OR=3.04 (2.99, 3.10)], 5-7 primary care visits[d] [OR=1.92 (1,89, 1.97)], female[d] [OR=0.95 (0.94, 0.97)], 18-30yrs old[d] [OR=0.30 (0.29, 0.36)], 31-40yrs olds[d] [OR=0.35 (0.35, 0.36)], 41-50yrs old[d] [OR=0.49 (0.48, 0.51)], 51-60yrs old[d] [OR=0.69 (0.68, 0.71)], neurological

© 2025 McIntosh SJ et al. *JAMA Network Open*.

									disorders[d] [OR= 1.76 (1.70, 1.81)], pain disorders[d] [OR=1.26 (1.21, 1.30)], migraine[d] [OR=1.26 (1.23, 1.29)], sleep disorders[d] [OR=0.95 (0.92, 0.99)], vestibular disorders[d] [OR=1.47 (1.45, 1.49)], TMJD[d] [OR=1.14 (1.05, 1.19)]
Meehan et al,[33] 2016	United States	mTBI clinic	Prospective cohort	N = 64[a,b]	21±2, (46.7%)	Consensus on concussion in sport	Sport-related (100%)	Initial (<21 days): (n=64)<br>Follow-up (32 ± 48 days): (n=64)	<u>1 month</u>: Initial PCSS[d] [OR=1.037 (1.011, 1.063)], PTA [OR=2.152 (0.561, 8.257)], initial number of symptoms [OR=0.927 (0.790, 1.102)]
Mehrolha-ssani et al,[26] 2020	Iran	ED	Prospective cohort	N = 364[a,b]	30 (IQR=17), 60%	Physician diagnosed, GCS 13-15	MVC (52.8%), violence (11.5%), sports (10.9%), falls (24.8%)	Initial (<24 hrs): (n=384)<br>Follow-up:<br>• 1 month (n=364)<br>• 3 months (n=364)	<u>1 month</u>: Non-MVC[d] [OR=8.78 (1.86, 41.35)], >1 initial symptom[d] [OR=3.46 (1.05, 11.42)], GCS <15 [n/a]
Ponsford et al,[27] 2012	Australia	ED	Prospective case-control	N=223[a], n=123[b]	31 [18-72], (26%)	LOC <30min, PTA <24 hrs, GCS 13-15	Assault (13.3%), MVC (40.9%), bicycle (20%), fall (12.5%), sport (8.3%), other (7.3%)	Initial (<48 hrs): (n=123)<br>Follow-up:<br>• 1 week (n=111)<br>• 3 months (n=90)	<u>3 months</u>: Anxiety (HADS) [d] [OR=1.42 (p=0.01)], age[d] [OR=1.07 (p=0.04)]
Richey et al,[20] 2020	United States	ED	Prospective cohort	N=447[a], n=359[c]	37.2 (IQR=13.6) , (39.8%)	VA/DoD	Pedestrian struck (12.3%), motor vehicle-traffic (30.4%), fall (23.4%), assault (19.2%), struck by/against (5.3%), bicycle (8.9%), other (0.6%)	Initial (unspecified): (n=359)<br>Follow-up:<br>• 1 month (n=259)<br>• 3 months (n=239)<br>• 6 months (n=221)	<u>6 months</u>: Age [OR=1.162 (0.666, 2.026)], age x time [OR=0.931 (0.831, 1.042)]
Rowe et al,[35] 2022	Canada	ED	Prospective cohort	N=250[a]	35 (IQR= 23-49), (52.4%)	WHO Criteria	Sports/recreat ion (25%), MVC (12%), assault (7%), fall (35%), work-related	Initial (<72 hrs): (n=250)<br>Follow-up:<br>• 30 days (n=222)<br>• 90 days (n=183)	<u>3 months</u>: Female[d] [OR=3.17 (1.60, 6.25)], age [OR=0.99 (0.97, 1.01)], eCPG [OR=0.60 (0.31, 1.15)], previous concussion history [OR=1.58 (0.82, 3.04)], history of sleep disorder[d] [OR=2.81 (1.22, 6.48)], sports/rec [OR=1.59 (0.64, 3.94)], MVC[d]

© 2025 McIntosh SJ et al. *JAMA Network Open*.

							(11%), other (10%)		[OR=4.64 (1.31, 16.5)], assault [OR=4.25 (0.93, 19.5)], work-related [OR=0.92 (0.30, 2.78)], other [OR=0.52 (0.15, 1.84)]
Rowe et al,[36] 2022	Canada	ED	Prospective cohort	N=248[a]	35 (IQR= 23-49), (52.4%)	WHO Criteria	Sports-related (25%), other (75%)	Initial (<72 hrs): (n=250)<br>Follow-up:<br>• 30 days (n=220)	1 month: Age [OR=1.01 (0.98, 1.03)], female[d] [OR=2.98 (1.52, 5.84)], history of depression or anxiety[d] [OR=3.21 (1.43, 7.23)], sports/rec [OR=1.66 (0.67, 4.06)], MVC[d] [OR=5.41 (1.34, 218)], assault [OR=3.95 (0.72, 21.8)], work-related [OR=1.87 (0.63, 5.55)], other [OR=0.74 (0.24, 2.23)]
Ryb et al,[21] 2014	United States	Trauma centre	Prospective cohort	N=180[a,b]	35.3±12.6, (36%)	GCS 13-15, LOC <30min, PTA/altered mental status	Not reported	Initial (<10 days): (n=180)<br>Follow-up:<br>• 3 months (n=109)<br>• 6 months (n=106)<br>• 12 months (n=102)	3 months: S100β>0.10 [OR=0.89 (0.30, 2.67)]<br>6 Months: S100β>0.10 [OR=0.42 (0.11, 1.56)]<br>12 Months: S100β>0.10 [OR=0.48 (0.12, 1.94)]
Sheedy et al,[32] 2009	Australia	ED	Prospective cohort	N=300[a], n=100[b,c]	33.6±12.7, (23.5%)	ACRM 1993	Assault (40.8%), low fall (24.5%), collision with/struck by object (23.5%), road accident (13.3%)	Initial (13.9 hrs): (n=98)<br>Follow-up (3 months): (n=78)	3 months: Occupational satisfaction [OR=1.13 (0.68, 1.89)], occupation [OR=1.40 (0.77, 2.56)], education level [OR=0.69 (0.25, 1.90)], immediate memory[d] [OR=0.53 (0.34, 0.84)], delayed memory[d] [OR=0.54 (0.32, 0.92)], headache[d] [OR=1.49 (1.14, 1.95)]
Varner et al,[37] 2021	Canada	ED	Secondary analysis of RCT	N=367[a], n=241[b]	33 (IQR=25-53), (61%)	Zurich Consensus Statement	Sport (8.3%), bike/MVC (27.4%), fall (35.3%), assault (6.2%), other (22.8%)	Initial (<48 hrs): (n=241)<br>Follow-up:<br>• 7 days (n=241)<br>• 14 days (n=241)<br>• 30 days (n=241)	1 month: Headache[d] [OR=7.7 (1.6, 37.8)], nausea [n/a], under influence of drugs/alcohol[d] [OR=5.9 (1.8, 19.4)], PTA [n/a], bike/MVC mechanism[d] [OR=2.9 (1.3, 6.0)], history of anxiety or depression[d] [OR=2.4 (1.2, 4.9)], numbness/tingling[d] [OR=2.4 (1.1, 5.2)]
Whittaker et al,[22] 2021	Canada	ED	Prospective cohort	N=92[a], n=73[b]	41.8, (57.5%)	GCS 13-15, LOC <20min, PTA <24hrs	Not reported	Initial (1-3 weeks): (n=92)<br>Follow-up (3 months): (n=73)	3 months: Symptoms [β=0.048 (SE=0.040)], timeline [β=0.207 (SE=0.111)], consequences[d] [β=0.207 (SE=0.064)]
Zuckerman et al,[23] 2016	United States	Sport (NCAA, ISP)	Prospective cohort	N=1507 [a,b]	Not reported, (31.2%)	Consensus on Concussion in Sport	Sport (100%)	Initial (unspecified): (n=1507)<br>Follow-up:<br>• 2 weeks (n=1507)	1 month: Helmet status [OR=1.43 (0.90, 2.29)], recurrent concussion[d] [OR=2.08 (1.28, 3.36)], number of symptoms [OR=0.88 (0.65, 1.19)], PTA [OR=1.34

© 2025 McIntosh SJ et al. *JAMA Network Open*.

								• 4 weeks (n=1507)	(0.67, 2.67)], retrograde amnesia[d] [OR=2.75 (1.34, 5.64)], difficulty concentrating[d] [OR=2.35 (1.23, 4.50)], dizziness [OR=1.35 (0.71, 2.56)], irritability [OR=0.97 (0.52, 1.82)], nausea/vomiting [OR=1.19 (0.69, 2.07)], loss of balance [OR=1.05 (0.59, 1.87)], visual disturbance [OR=1.31 (0.76, 2.24)], light sensitivity[d] [OR=1.97 (1.09, 3.57)], noise sensitivity [OR=1.53 (0.86, 2.74)], insomnia[d] [OR=2.19 (1.30, 3.68)], drowsiness [OR=1.56 (0.88, 2.77)]
**Post-concussion symptom severity (RPQ, RHIFQ, PCSC, Neurobehavioral symptom inventory)**									
Brett et al,[39] 2021	United States	ED	Prospective cohort	N=1757[a], n=1021[b]	29.9±17.0, (32.6%)	GCS 13-15, CT-	MVC (57.6%), fall (26.1%), assault/violence (6.9%), other (9.4%)	Initial (<24 hrs): (n=1757)<br>Follow-up:<br>• 2 weeks (n=1537)<br>• 6 months	6 months: Model 1 (Age, Sex, Race, Education, Psychiatric history, TBI severity) [OR=0.03 (-3.28, 3.34)], Model 2 (Age, Sex, Race, Education, Psychiatric history, TBI severity, and Neurobehavioural latent class) [OR=-1.29 (-4.23, 1.64)]
Heitger et al,[40] 2007	New Zealand	ED	Prospective cohort	N=74[a], n=37[c]	29.1±12.7, (35%)	GCS 13-15, PTA <24hrs, LOC <15min	MVC (24%), bike (22%), rugby (16%), falls (16%), horse riding (11%), netball (5%), soccer (3%), rollerblading (3%)	Initial (<1 week): (n=37)<br>Follow-up:<br>• 3 months (n=37)<br>• 6 months (n=37)<br>• 12 months (n=31)	3 months: RPQ: IQ [β=-0.38, p=0.030]<br>6 months: RPQ: IQ [β=-0.31, p=0.085], LOC [β=-0.26, p=0.140]; RHIFQ: IQ [β=-0.38, p=0.034]<br>12 months: RPQ: IQ [β=-0.42, p=0.033]; RHIFQ: IQ [β=-0.34, p=0.094]
Keatley et al,[44] 2023	United States	ED	Prospective cohort	N=310[a,] n=253[b]	44.4±19.3, (38.4%)	VA/DoD	Fall from height (11.1%), MVC (26.1%), pedestrian struck (9.1%), MCC (7.9%), assault (18.6%), bicycle (1.2%), struck by or against (4.7%)	Initial (<24 hrs): (n=246)<br>Follow-up:<br>• 1 month (n=239)<br>• 3 months (n=238)<br>• 6 months (n=253)	6 months: Minimal PCS: Age [RRR=1.00 (0.98, 1.03)], female sex [RRR=0.98 (0.37, 2.58)], Black/African American [RRR=0.99 (0.39, 2.56)], Hispanic/Latin [RRR=1.70 (0.32, 9.00)], years of education[d] [RRR=1.22 (1.04, 1.42)], married/partner [RRR=0.97 (0.38, 2.49)], history of depression [RRR=0.15 (0.02, 1.18)], prior concussion [RRR=0.23 (0.05, 1.04)], employment status [RRR=1.00 (0.41, 2.45)], work-related injury [RRR=0.53 (0.06, 4.36)], drug/alcohol use within 24

© 2025 McIntosh SJ et al. *JAMA Network Open*.

									hrs of study enrollment [RRR=1.05 (0.41, 2.70)], altered mental state [RRR=1.51 (0.58, 3.97)], LOC[d] [RRR = 0.29 (0.11, 0.78)], PTA [RRR=0.58 (0.22, 1.59)]<br>High acute/decreasing PCS: age [RRR=0.99 (0.98, 1.01)], female sex [RRR=1.71 (0.95, 3.08)], Black/African American [RRR=1.36 (0.76, 2.43)], Hispanic/Latin [RRR=0.44 (0.09, 2.24)], years of education [RRR=1.00 (0.90, 1.12)], married/partner [RRR=1.03 (0.57, 1.87)], history of depression[d] [RRR=2.69 (1.45, 4.98)], history of other psychiatric diagnoses [RRR=1.51 (0.78, 2.90)], prior concussion [RRR=1.04 (0.56, 1.94)], employment status [RRR=1.46 (0.82, 2.59)], work-related injury [RRR=1.25 (0.46, 3.39)], drug/alcohol use within 24 hrs of study enrollment [RRR=0.90 (0.49, 1.65)], altered mental state [RRR=0.95 (0.54, 1.70)], LOC[d] [RRR=0.79 (0.38, 1.67)], PTA [RRR=1.26 (0.60, 2.63)]<br>High acute/increasing PCS: age [RRR=0.98 (0.96, 1.00)], female sex[d] [RRR=2.82 (1.29, 6.19)], Black/African American[d] [RRR=2.52 (1.07, 5.93)], Hispanic/Latin[d] [RRR=3.82 (1.14, 12.76)], years of education [RRR=0.93 (0.80, 1.09)], married/partner [RRR=0.47 (0.19, 1.18)], history of depression[d] [RRR=2.64 (1.18, 5.92)], history of other psychiatric diagnoses[d] [RRR=2.59 (1.14, 5.88)], prior concussion [RRR=1.32 (0.59, 2.98)], employment status [RRR=0.64 (0.29, 1.39)], work-related injury [RRR=0.72 (0.15, 3.52)], drug/alcohol use within 24 hrs of study enrollment [RRR=1.97 (0.91, 4.29)], altered mental state [RRR=2.15 (0.89, 5.17)], LOC[d] [RRR=0.73 (0.28, 1.93)], PTA [RRR=0.47 (0.20, 1.08)]
McCauley et al,[38] 2013	United States	ED	Prospective cohort	N=75[a], n=46[b,c]	30.6±9.6, (26.1%)	VA/DoD	Assault (5.3%), auto-pedestrian	Initial (19.6±9.4 hrs): (n=46)<br>Follow-up:	<u>1 month</u>: Age [β=-0.06 (SE=0.16)], Sex [β=0.17 (SE=3.32)], Group [β = 0.28

© 2025 McIntosh SJ et al. *JAMA Network Open*.

							(4.0%), blow to head (2.7%), fall (17.3%), MVC (30.7%), sports (1.3%)	• 1 week (n=46)<br>• 1 month (n=46)	(SE=3.03)], CES-D [β=0.45 (SE=0.22)], CD-RISC [β=0.30 (SE=0.09)]
Ponsford et al,[43] 2000	Australia	ED	Prospective case-control	N=137[a], n=84[c]	26.4±13.9, (38%)	ACRM 1993	MVC (25%), fall (19%), cycling (14%), assault (11%), sports (23%), other (8%)	Initial (<1 week): (n=84)<br>Follow-up (3 months): (n=84)	<u>3 months</u>: Neurological/psychiatric problems [7.8% variance], student [7.7% variance], Holmes-Rahe score [6.5% variance]
Ponsford et al,[41] 2019	Australia	ED	Prospective cohort	N=536 [a], n=343[b]	54±21, (45.5%)	GCS 13-15	Fall (51.9%), violence or assault (14.9%), sports-related (8.2%), road traffic incident (6.1%), other (19%)	Initial (<24 hrs): (n=343)<br>Follow-up (130-320 days): (n=343)	Model 1: Age [OR=0.98 (0.97, 0.99)], male [OR=1.53 (0.95, 2.47)]<br>Model 2: Psychological history[d] [OR=2.75 (1.68, 4.50)], time post-injury [O=1.00 (0.99, 1.01)], history of illicit substance use [OR=1.26 (0.63, 2.50)], history of alcohol use [OR=1.83 (0.83, 4.01)]<br>Model 3: GCS [OR=0.78 (0.25, 2.45)], unknown LOC[d] [OR=2.52 (1.35, 4.67)], positive LOC[d] [OR=1.94 (1.22, 3.10)], no other injury [OR=1.14 (0.66, 1.97)], unknown if other injury [OR=0.73 (0.48, 1.10)]<br>Model 4: Recall receiving information [OR=2.35 (1.18, 4.68)], no recall of receiving information [OR=2.65 (1.52, 4.63)]<br>Model 5: Age [OR=0.99 (0.98, 1.00)], psychological history [OR=2.99 (1.83, 4.89)], unknown LOC [OR=2.47 (1.56, 3.92)], positive LOC [OR=2.0 (1.07, 3.71)], recall receiving information [OR=2.01 (0.92, 4.40)], no recall of receiving information [OR=2.34 (1.27, 4.30)]
Preiss-Farzenagan et al,[45] 2009	United States	NIH-funded TBI registry	Prospective cohort	N=260[a], n=78[c]	Males: 36.9±13.3; Females 30.1±12, (40%)	ACRM 1993	Sport-related (100%)	Initial (unspecified): (n=260)<br>Follow-up (3 months): (n=215)	<u>3 months</u>: RPQ-total: Female[d] [OR=2.57 (1.09, 6.08)], age [OR=1.02 (0.99, 1.06)]; RPQ-3: Female [OR=2.97 (1.19, 7.41)], age [OR=1.02 (0.98, 1.06)], helmeted sports [OR=0.53 (0.14, 2.00)]; RPQ-13: Female

© 2025 McIntosh SJ et al. *JAMA Network Open*.

									[OR=2.54 (1.01, 6.38)], age [OR=1.02 (0.98, 1.06)]
Remigio-Baker et al,[51] 2020	United States	Military medical unit	Prospective cohort	N=116[a], n=111[b]	24 (IQR=21-29), (9.9%)	VA/DoD	Not reported	Initial (<72 hrs): (n=116)<br>Follow-up:<br>• 1 week (n=111)<br>• 3 months (n=111)<br>• 6 months (n=111)	6 months: Total [β=-0.06 (-0.11, -0.01)], cognitive items[d] [β=-0.05 (-0.10, -0.01)], vestibular/balance items[d] [β=-0.06 (-0.11, -0.01)], somatosensory items[d] [β=-0.05 (-0.10, -0.003)], affective items[d] [β=-0.05 (-0.09, -0.003)]
Sheedy et al,[32] 2009	Australia	ED	Prospective cohort	N=135[a], n=95[b,c]	35.6 [10-86], (42.1%)	GCS 13-15, PTA/altered mental status <5min	Not reported	Initial (<22 hrs): (n=95)<br>Follow-up:<br>• 30 days (n=75)<br>• 90 days (n=65)	3 months: SNTF- vs. SNTF+ [p >0.5]
**Quality of life (QOLIBRI, SF-36)**									
Brett et al,[39] 2021	United States	ED	Prospective cohort	N=1757[a], n=1021[b]	29.9±17.0, (32.6%)	GCS 13-15, CT-	MVC (57.6%), fall (26.1%), assault/violence (6.9%), other (9.4%)	Initial (<24 hrs): (n=1757)<br>Follow-up:<br>• 2 weeks (n=1537)<br>• 6 months	6 months: Model 1 (Age, Sex, Race, Education, Psychiatric history, TBI severity) [OR=0.01 (-0.22, 0.24)], Model 2 (Age, Sex, Race, Education, Psychiatric history, TBI severity, and Neurobehavioural latent class) [OR=0.59 (0.34, 0.84)]
Heitger et al,[40] 2007	New Zealand	ED	Prospective cohort	N=74[a], n=37[c]	29.1±12.7, (35%)	GCS 13-15, PTA <24hrs, LOC <15min	MVC (24%), bike (22%), rugby (16%), falls (16%), horse riding (11%), netball (5%), soccer (3%), rollerblading (3%)	Initial (<1 week): (n=37)<br>Follow-up:<br>• 3 months (n=37)<br>• 6 months (n=37)<br>• 12 months (n=31)	3 months: Physical summary: IQ [β=0.46, p=0.008]; mental summary: GCS [β=-0.33, p=0.064]<br>6 months: Physical summary: IQ [β=0.37, p=0.038]; mental summary: IQ [β=0.32, p=0.075]<br>12 months: Physical summary: IQ [β=0.40, p=0.044]; mental summary: IQ [β=0.32, p=0.075]
**Life satisfaction (SWLS)**									
Brett et al,[39] 2021	United States	ED	Prospective cohort	N=1757[a], n=1021[b]	29.9±17.0, (32.6%)	GCS 13-15, CT-	MVC (57.6%), fall (26.1%), assault/violence (6.9%), other (9.4%)	Initial (<24 hrs): (n=1757)<br>Follow-up:<br>• 2 weeks (n=1537)<br>• 6 months	6 months: Model 1 (Age, Sex, Race, Education, Psychiatric history, TBI severity) [OR=1.62 (-0.12, 3.35)], Model 2 (Age, Sex, Race, Education, Psychiatric history, TBI severity, and Neurobehavioural latent class) [OR=2.49 (0.83, 4.160)]
**Functional outcome (GOS-E)**									
Brett et al,[39] 2021	United States	ED	Prospective cohort	N=1757[a], n=1021[b]	29.9±17.0, (32.6%)	GCS 13-15, CT-	MVC (57.6%), fall (26.1%),	Initial (<24 hrs): (n=1757)<br>Follow-up:	6 months: Model 1 (Age, Sex, Race, Education, Psychiatric history, TBI

© 2025 McIntosh SJ et al. *JAMA Network Open*.

							assault/violence (6.9%), other (9.4%)	• 2 weeks (n=1537)<br>• 6 months	severity) [OR=0.56 (0.30, 0.83)], Model 2 (Age, Sex, Race, Education, Psychiatric history, TBI severity, and Neurobehavioural latent class) [OR=0.59 (0.34, 0.84)]
Richey et al,[20] 2020	United States	ED	Prospective cohort	N=447[a], n=359[c]	37.2 (IQR=13.6), (39.8%)	VA/DoD	Pedestrian struck (12.3%), motor vehicle-traffic (30.4%), fall (23.4%), assault (19.2%), struck by/against (5.3%), bicycle (8.9%), other (0.6%)	Initial (unspecified): (n=359)<br>Follow-up:<br>• 1 month (n=259)<br>• 3 months (n=239)<br>• 6 months (n=221)	<u>6 months</u>: Age[d] [OR=7.554 (3.088, 18.479)], age x time[d] [OR=0.790 (0.675, 0.925)]
**Time to symptom resolution/Symptom duration**									
Chandran et al,[46] 2024	United States	Sport (NCAA, ISP)	Prospective cohort	N=1709	Not reported, (29.2%)	Consensus on Concussion in Sport	Sport (100%); player contact (67.2%), other contact (29.3%)	Initial (<14 days)<br>Follow-up (>28 days)	<u>1 Month</u>: Men: Cross-dependent symptoms (difficulty concentrating, irritability, sensitivity to noise, headache, and drowsiness) [d] [OR=1.30 (1.15, 1.47)], total number of symptoms[d] [OR=1.16 (1.09, 1.23)]; Women: cross-dependent symptoms (difficulty concentrating, irritability, sensitivity to noise, and hyperexcitability) [d] [OR=1.21 (1.01, 1.44)], total number of symptoms[d] [OR=1.15 (1.05, 1.25)]
Howell et al,[47] 2016	United States	Sport clinic	Prospective cohort	N=364[a], n=30[c]	Not reported, (11%)	Consensus on Concussion in Sport	Sport-related (89.6%), Non-sport-related (10.4%)	Initial (<21 days): (n=364)<br>Follow-up (at symptom recovery [48.9±76.0 days]): (n=248)	<u>48.9±76.0 days</u>: Age [HR=0.674 (0.330, 1.375)], sex [HR=2.937 (0.539, 16.018)], initial symptom severity[d] [HR=0.940 (0.886, 0.997)], number of previous concussions [HR=0.607 (0.259, 1.421)], LOC [HR=0.195 (0.012, 3.147)], PTA [HR=0.115 (0.006, 2.402)], prior treatment for headaches [HR=3.585 (0.056, 228.28)], initial period of physical rest [HR=5.413 (0.452, 64.876)], physical activity post-injury [HR=0.998 (0.978, 1.018)]
**Post-traumatic stress disorder (ASDS, PCL-C)**									

© 2025 McIntosh SJ et al. *JAMA Network Open*.

Gil et al,[49] 2005	Israel	Surgical ward	Prospective cohort	N=198 [a], n=120[b]	31.4±2.7, (42%)	GCS 13-15	Traffic-related (90%)	Initial (<24 hrs): (n=120)<br>Follow-up (6 months): (n=120)	6 months: Memory of traumatic event[d] [OR=2.2 (1.0, 10.1)], acute PTSD symptoms (clinician administered PTSD scale)[d] [OR=5.3 (1.1, 9.3)], acute PTSD symptoms (post-traumatic stress scale) [d] [OR=5.2 (1.0, 9.4)], depressive symptoms[d] [OR=5.1 (1.0, 9.2)], anxiety symptoms[d] [OR=4.9 (1.0, 9.1)], age [n/a], history of psychiatric disorder[d] [OR=3.7 (1.1, 8.9)], sex [n/a]
McCauley et al,[38] 2013	United States	ED	Prospective cohort	N=75[a], n=46[b,c]	30.6±9.6, (26.1%)	VA/DoD	Assault (5.3%), auto-pedestrian (4.0%), blow to head (2.7%), fall (17.3%), MVC (30.7%), sports (1.3%)	Initial (19.6±9.4 hrs): (n=46)<br>Follow-up:<br>• 1 week (n=46)<br>• 1 month (n=46)	1 month: ASDS: Age [β=-0.14 (SE=0.21)], sex [β = 0.18 (SE=4.31)], group[d] [β = 0.28 (SE=3.93)], CES-Dv [β=0.52 (SE=0.28)], CD-RISC[d] [β=0.31 (SE=0.11)]; PCL-C: Age [β=-0.15 (SE=0.18)], sex [β=0.12 (SE=3.63)], group[d] [β=0.27 (SE=3.31)], CES-D[d] [β=0.48 (SE=0.24)], CD-RISC[d] [β=0.28 (SE=0.1)]
**Post-injury clinician visits**									
Kruse et al,[50] 2018	United States	Mayo Clinic	Retrospective chart review	N=120[a]	Treatment: 47.6±8.3, (26.7%);<br>No treatment: 45.5±13.5, (43.3%)	ICD-9 codes	Fall (36.7%)	Initial (unspecified): (n=120)<br>Follow-up:<br>• 3 months (n=120)<br>• 6 months (n=120)<br>• 12 months (n=120)	3 months: Pharmacologic treatment for depression at time of injury[d] [β=-1.05 (-1.28, -0.83)], age [β= -0.00 (-0.01, 0.01)], male[d] [β=-0.32 (-0.53, -0.10)], college education[d] [β=0.21 (0.01, 0.40)], fall [β=-0.12 (-0.32, 0.08)], probable injury severity classification [β=0.18 (-0.03, 0.38)]<br>6 months: Pharmacologic treatment for depression at time of injury[d] [β=-1.51 (-1.70, -1.32)], age[d] [β=0.02 (0.01, 0.02)], male [d] [β=-0.21 (-0.36, -0.06)], college education [d] [β=0.29 (0.14, 0.44)], fall [d] [β=-0.38 (-0.53, -0.22)], probable injury severity classification [d] [β=0.30 (0.15, 0.45)]<br>12 months: Pharmacologic treatment for depression at time of injury [d] [β=-1.76 (-1.93, -1.59)], age [d] [β=0.02 (0.01, 0.02)], male [β=-0.11 (-0.24, 0.01)], college education[d] [β=0.18 (0.06, 0.31)], fall [d] [β=-0.20 (-0.33, -0.07)], probable injury

© 2025 McIntosh SJ et al. *JAMA Network Open*.

									severity classification [d] [β=0.27 (0.14, 0.40)]
**Return to work**									
Nolin et al,[48] 2006	Canada	ED	Prospective cohort	N=110[a]	RTW: 34.6±14.7, (16.7%); No RTW: 31.0±12.1, (22.6%)	ACRM 1993	Not reported	Initial: (unspecified): (n=110)<br>Follow-up:<br>• 1-3 months (n=108)<br>• 12-36 months (n=85)	12-36 months: Age [β=0.02 (SE=0.02)], sex [β=0.28 (SE=0.72)], GCS [β=1.66 (SE=0.95)], duration of PTA [β=0.00 (SE=0.00)], duration of retrograde amnesia [β=0.00 (SE=0.03)], initial symptoms [β=-0.06 (SE=0.23)], symptoms at follow-up [d] [β=-0.17 (SE=0.04)], time since injury [β=0.04 (SE=0.05)], PASAT1 [β=0.28 (SE=0.06)], PASAT2 [β=-0.04 (SE=0.06)], Stroop1 [β=0.04 (SE=0.06)], Stroop2 [β = 0.01 (SE=0.06)], Stroop3 [β=-0.01 (SE=0.03)], Stroop4 [β=-0.03 (SE=0.03)], CVLT total number of words [β=0.05 (SE=0.07)], CVLT immediate recall [β = -0.07 (SE=0.31)], CVLT delayed recall [β = 0.02 (SE=0.34)], CVLT recognition [β = 0.40 (SE=0.32)]
Ryb et al,[21] 2014	United States	Trauma centre	Prospective cohort	N=180[a,b]	35.3±12.6, (36%)	GCS 13-15, LOC <30min, PTA/altered mental status	Not reported	Initial (<10 days): (n=180)<br>Follow-up:<br>• 3 months (n=109)<br>• 6 months (n=106)<br>• 12 months (n=102)	3 months: S100β>0.10 [OR=2.04 (0.64, 6.54)]<br>6 Months: S100β>0.10 [OR=7.60 (0.59, 98.26)]<br>12 Months: S100β>0.10 [OR=10.29 (0.92, 114.84)]
**Physical activity**									
Remigio-Baker et al,[51] 2020	United States	Military medical unit	Prospective cohort	N=116[a], n=111[b]	24 (IQR=21-29), (9.9%)	VA/DoD	Not reported	Initial (<72 hrs): (n=116)<br>Follow-up:<br>• 1 week (n=111)<br>• 3 months (n=111)<br>• 6 months (n=111)	6 months: Received education: Total [d] [β=0.11 (0.06, 0.17)], lifestyle items [d] [β=0.06 (0.01, 0.11)], physical items [d] [β=0.12 (0.07, 0.18)], vestibular/balance items [d] [β=0.10 (0.04, 0.16)], military-specific items [d] [β=0.11 (0.05, 0.17)]; No education: Total [β=-0.05 (-0.12, 0.03)], lifestyle items [d] [β =-0.10 (-0.16, -0.03)], physical items [β=-0.03 (-0.10, 0.05)], vestibular/balance itmes [β=-0.01 (-0.10, 0.07)], military-specific items [β=-0.001 (-0.09, 0.08)]
**Depressive symptoms (PHQ-9)**									

© 2025 McIntosh SJ et al. *JAMA Network Open*.

Richey et al,[20] 2020	United States	ED	Prospective cohort	N=447[a], n=359[c]	37.2 (IQR=13.), (39.8%)	VA/DoD	Pedestrian struck (12.3%), motor vehicle-traffic (30.4%), fall (23.4%), assault (19.2%), struck by/against (5.3%), bicycle (8.9%), other (0.6%)	Initial (unspecified): (n=359)<br>Follow-up:<br>• 1 month (n=259)<br>• 3 months (n=239)<br>• 6 months (n=221)	6 months: Age [OR=0.658 (0.386, 1.123)], age x time [d] [OR=0.870 (0.777, 0.974)]
**Other undefined poor outcome**									
Topolovec-Vranic et al,[52] 2011	Canada	ED	Prospective cohort	N=141[a,b]	39.4 [19-65], (36.9%)	GCS 13-15	MVC (37%), assault (6%), sport (1%), fall (45%), pedestrian struck (4%), other (7%)	Initial (<4 hours): (n=141)<br>Follow-up:<br>• 3 days (n=115)<br>• 1 week (n=113)<br>• 6 weeks (n=95)	6 weeks: Sex[d] [OR=0.20 (0.05, 0.81)], LOC [d] [OR=8.11 (1.95, 33.74)], day 3 RPQ [d] [OR=1.13 (1.05, 1.21)], NSE level [d] ≥14.6 ug/L [OR=5.32 (1.42, 19.91)]

[a] Total sample enrolled
[b] Sample in analysis
[c] Subgroup of eligible participants
[d] Statistically significant in seed study

Abbreviations: ACRM, American Congress of Rehabilitation Medicine; ASDS, Acute Stress Disorder Scale; BIPQ, Brief Illness Perception Questionnaire; BRIQ, Behavioural Response to Illness Questionnaire; CD-RISC, Connor-Davidson Resilience Scale; CES-D, Center for Epidemiologic Studies Depression Scale; CT, computed tomography; eCPG, electronic clinical practice guidelines; DoD, Department of Defense; ED, Emergency department; GCS, Glasgow Coma Scale; GOSE, Glasgow Outcome Scale Extended; HADS, Hospital Anxiety and Depression Scale; LOC, loss of consciousness; MCC, motor cycle collision; mTBI: mild traumatic brain injury; MVC, motor vehicle collision; OHIP, Ontario Health Insurance Plan; NSE, neuron-specific enolase; PCS; PCSS; PTA, post-traumatic amnesia; PTSD, post-traumatic stress disorder; QoL, Quality of life; QOLIBRI-OS, Quality of Life After Brain Injury-Overall Scale; RPQ, Rivermead post-concussion questionnaire; RHIFQ, Rivermead Head-Injury Follow-Up Questionnaire; RSRRS: The Revised Social Readjustment Rating Scale; SCAT3, Sport Concussion Assessment Tool 3; SF-36, 36-Item Short Form Survey; SWLS, Satisfaction with life scale; TBI, traumatic brain injury; TMJD, Temporomandibular joint dysfunction disorder; UPSIT, University of Pennsylvania Smell Identification Test; VA, Veterans Affairs;

© 2025 McIntosh SJ et al. *JAMA Network Open*.

eTable 4. QUIPs Risk of Bias Assessment

Author, Year	1. Study population	2. Study attrition	3. Prognostic factor measurement	4. Outcome measurement	5. Study confounding	6. Statistical analysis & reporting	Overall
Bazarian et al,[25] 1999	L	M	L	L	L	H	Moderate
Brett et al,[39] 2021	M	H	L	L	H	L	High
Caplain et al,[28] 2017	H	L	M	L	H	L	High
Chandran et al,[46] 2024	M	L	L	L	L	L	Low
Dischinger et al,[29] 2009	M	H	L	L	L	L	Moderate
Eskridge et al,[34] 2013	L	L	L	L	M	L	Low
Faux et al,[30] 2011	M	H	L	L	M	L	High
Foster et al,[19] 2022	L	H	L	L	L	L	Moderate
Gil et al,49 2005	H	H	L	L	M	L	High
Heitger et al,[40] 2007	H	H	L	L	M	L	High
Hou et al,[31] 2012	L	H	L	L	L	L	Moderate
Howell et al,[47] 2016	M	H	L	L	M	L	Moderate
Keatley et al,[44] 2023	L	M	L	L	L	L	Low
Kruse et al,[50] 2018	L	L	L	L	L	L	Low
Langer et al,[24] 2021	L	L	L	L	M	L	Low
McCauley et al,[38] 2013	H	L	L	L	L	L	Moderate
Meehan et al,[33] 2016	M	L	L	L	M	L	Moderate
Mehrolhassani et al,[26] 2020	L	L	L	L	H	L	Moderate
Nolin et al,[48] 2006	M	H	L	L	L	L	Moderate
Ponsford et al,[43] 2000	H	L	L	L	L	L	Moderate
Ponsford et al,[27] 2012	L	H	M	L	H	L	High
Ponsford et al,[41] 2019	H	H	L	L	M	L	High
Preiss-Farzenagan et al,[45] 2009	L	M	L	L	L	L	Low
Remigio-Baker et al,[51] 2020	M	L	L	L	L	L	Low
Richey et al,[20] 2020	L	H	L	M	H	M	High
Rowe et al,[35] 2022	L	M	L	L	L	L	Low
Rowe et al,[36] 2022	L	H	L	L	H	L	High
Ryb et al,[21] 2014	H	H	L	L	L	L	High
Sheedy et al,[32] 2009	M	H	M	L	M	L	High
Siman et al,[42] 2020	H	H	L	L	M	L	High
Topolovec-Vranic et al,[52] 2011	H	H	L	M	M	L	High
Varner et al,[37] 2021	M	H	L	L	H	L	High
Whittaker et al,[22] 2007	M	H	L	L	H	H	High
Zuckerman et al,[23] 2016	L	L	L	L	M	L	Low

© 2025 McIntosh SJ et al. *JAMA Network Open*.

eFigure 2. Meta-Analysis of Factors Associated With Persisting Symptoms After Concussion (PSAC) at 1 Month

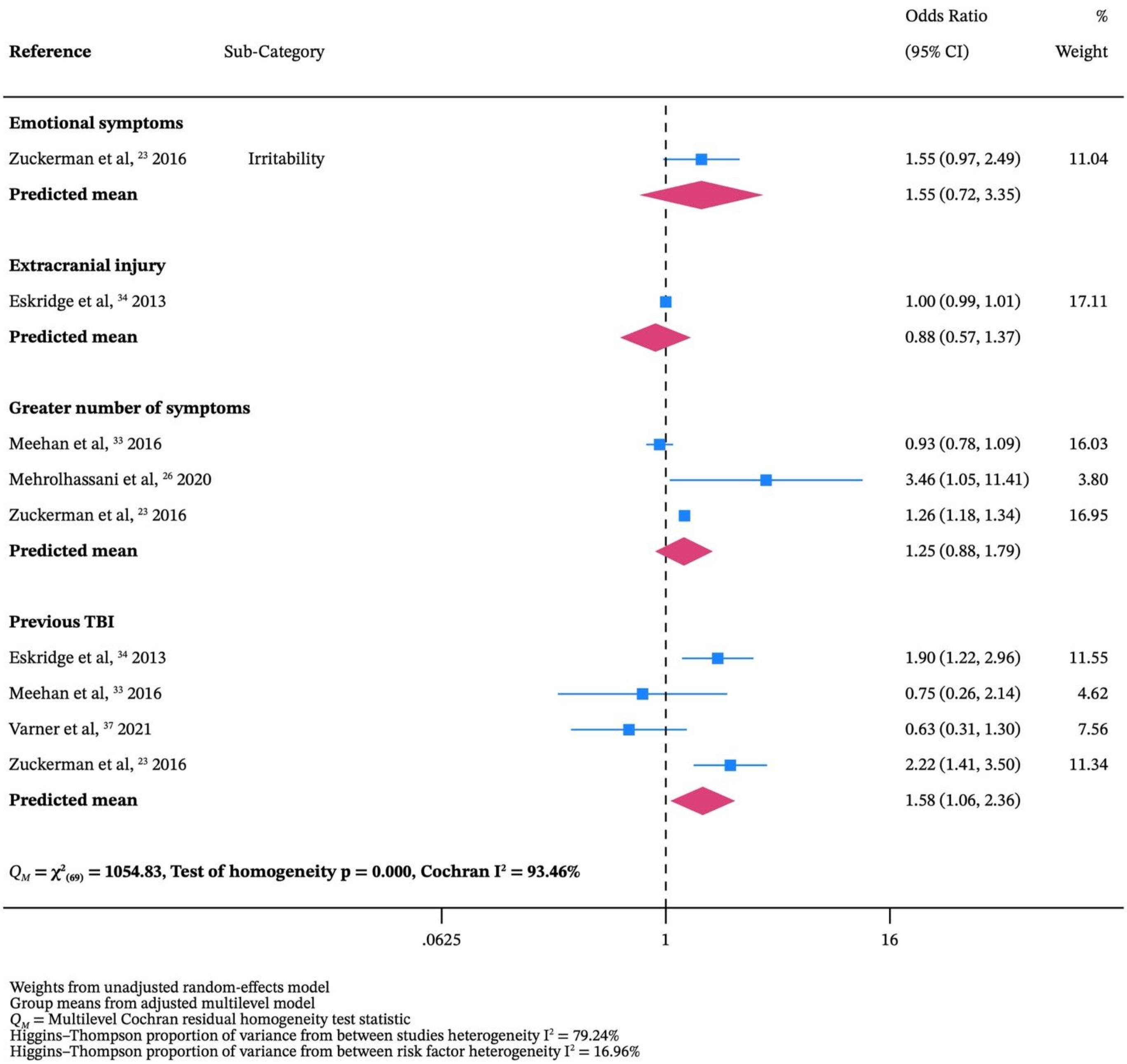

Weights are from the unadjusted random-effects model. Group means are from the adjusted multilevel model. Higgins-Thompson proportion of variance from between-studies heterogeneity, I2 = 79.24%; Higgins-Thompson proportion of variance from between-risk factor heterogeneity, I2 = 16.96%. AOR indicates adjusted odds ratio; Q_M, multilevel Cochran residual homogeneity test statistic; TBI, traumatic brain injury.

© 2025 McIntosh SJ et al. *JAMA Network Open*.
