# Supplementary material for: Factors Associated With Persisting Symptoms After Concussion in Adults With Mild TBI: A Systematic Review and Meta-Analysis
Source: JAMA Netw Open. 2025 Jun 18;8(6):e2516619. doi: 10.1001/jamanetworkopen.2025.16619 (PMC12177663; doi:10.1001/jamanetworkopen.2025.16619)
Supplement: Supplement 2. — Data Sharing Statement [file jamanetwopen-e2516619-s002.pdf]

Data Sharing Statement

McIntosh. Factors Associated With Persisting Symptoms After Concussion in Adults With Mild TBI. *JAMA Netw Open*. Published June 18, 2025. doi:10.1001/jamanetworkopen.2025.16619

Data
Data available: No
